# The effect of non-pharmacological prenatal interventions on fear of childbirth: an overview of systematic reviews and meta-analysis

**DOI:** 10.1186/s12888-024-05870-5

**Published:** 2024-06-04

**Authors:** Zohreh Alizadeh-Dibazari, Mahsa Maghalain, Mojgan Mirghafourvand

**Affiliations:** 1 Department of Midwifery, Women’s Reproductive and Mental Health Research Centre, Tabriz Medical Sciences, Islamic Azad University, Tabriz, Iran; 2 Department of Midwifery, Faculty of Medical Science, Tabriz Medical Sciences, Islamic Azad University, Tabriz, Iran; 3 Students Research Committee, Department of Midwifery, Faculty of Nursing and Midwifery, Tabriz University of Medical Sciences, Tabriz, Iran; 4 Social Determinants of Health Research Center, Tabriz University of Medical Sciences, Tabriz, Iran

**Keywords:** Fear of childbirth, FoC, Psychological intervention, Prenatal education, Distraction technique, Enhanced care, Overview of systematic reviews

## Abstract

**Background:**

During pregnancy and childbirth, alongside positive feelings, women undergo feelings such as fear of childbirth (FoC) and worry about its consequences, which could leave negative effects on the mother and her child during pregnancy, delivery, and postpartum. The study was carried out to determine the effectiveness of prenatal non-pharmacological interventions on reducing the FoC.

**Methods:**

The protocol of the study was registered in PROSPERO (ID: CRD42023468547). PubMed, Web of Science, Cochrane, Scopus, SID (Scientific Information Database) and Google Scholar search engine databases were systematically searched until July 27, 2023 with no limitation of time and limited to Persian and English studies in order to perform this overview. Certainty of evidence was assessed using GRADE, methodological quality using AMSTAR 2 and reporting quality using PRISMA score. Meta-analysis was performed on the data extracted from the original trials to evaluate the effect of different interventions on reducing the FoC. Sub-group analysis and meta-regression models were used to examine high heterogeneity, and sensitivity analysis was used to eliminate the effect of high risk of bias studies on the study findings.

**Results:**

Overall, 15 systematic reviews (SRs) were included in the overview, among which meta-analysis was performed in 9 studies. Considering methodological quality, these SRs were in low to critically low status and had relatively complete reports regarding reporting quality. Meta-analysis findings indicated that psychological interventions (SMD -2.02, 95% CI -2.69 to -1.36, 16 trials, 1057 participants, I^2^ = 95%) and prenatal educations (SMD -0.88, 95% CI -1.16 to -0.61, 4 trials, 432 participants, I^2^ = 72.8%) cause a significant reduction in FoC relative to prenatal usual cares with low certainty of evidence. Distraction techniques lead to a significant reduction in FoC relative to prenatal usual care with high certainty of evidence (SMD -0.75, 95% CI -1.18 to -0.33, 4 trials, 329 participants, I^2^ = 69%), but enhanced cares do not result in a significant decrease FoC relative to prenatal usual care with very low certainty of evidence (SMD -1.14, 95% CI -2.85 to 0.58, 3 trials, 232 participants, I^2^ = 97%).

**Conclusions:**

Distraction techniques are effective in reducing FoC. Regarding the effect of psychological interventions and prenatal educations on the reduction of FoC, the findings indicated that the interventions may result in the reduction of FoC. Very uncertain evidence showed that enhanced cares are not effective in reducing the FoC.

**Supplementary Information:**

The online version contains supplementary material available at 10.1186/s12888-024-05870-5.

## Background

Childbirth is an exciting incidence for mothers. Nonetheless, fear and worry about childbirth and its consequences can cause more anxiety in the mother besides the positive feelings that the mother feel [1]. Fear of childbirth (FoC) generally refers to the feeling of fear, anxiety or worry about pregnancy and childbirth [2] and encompasses some fearful thoughts and feelings about childbirth, ranging from normal fears to severe ones (fear that affects daily activities) [3]. Tocophobia is defined as severe FoC. Women with tocophobia delay pregnancy because of the FoC, particularly natural childbirth, and in most cases, they request a cesarean delivery [4, 5]. Mild FoC is seen in 80 percent of pregnant women, moderate FoC in 20 percent of pregnant women, and severe FoC in 6-10 percent of pregnant women, affecting their daily life. Further, 13% of non-pregnant women delay pregnancy or are not willing to give birth because of FoC [6]. Many studies differentiate between primary and secondary FoC. Primary FoC is present prior to childbirth for the first time, yet secondary FoC starts after a negative birth experience [7].

The global prevalence of tocophobia is estimated to be 14 percent that differs significantly from one region to another [4]. These differences could be because of general ignorance about FoC and its risk factors that results in the use of various measurement tools or even different cut points in the same tool [2]. Moreover, the prevalence of FoC could vary in various cultures and countries [8].

Many reasons have been reported for FoC, which are young age of the mother, low education level, nulliparity, previous negative experiences, fear of pain caused by childbirth, fear of unsuccessful childbirth, existing psychological problems like lack of self-confidence about the ability for childbirth, low social support, history of anxiety or depression, unpleasant sexual experiences and concern about the child's health [8, 9]. However, different studies have stated the fear of natural childbirth pain as the main reason for FoC. Fears during pregnancy may predict pain and discomfort during childbirth [10].

FoC has negative effects on prenatal, delivery and postpartum periods [11]. Most probably, FoC leads to complications like high blood pressure, pre-eclampsia, low birth weight, premature delivery [12], the ineffective uterine contractions, higher level of labor pain, prolonged labor, instrumental vaginal delivery, emergency cesarean delivery [13], postpartum anxiety and depression, post-traumatic stress disorder (PTSD), ineffective mother-child relationships, and emotional or behavioral problems in childhood [14].

The purpose of FoC management is to help the woman to accept the uncertainties associated with childbirth to control the pregnancy, reduce the anxiety associated with childbirth and increase the rate of vaginal birth (VB) [15]. Several trials have been carried out on the effect of various interventions on reducing FoC during pregnancy and postpartum. These interventions include theory-based childbirth educations [16], childbirth preparation classes [17], theory-based counseling [18], cognitive-behavioral therapy (CBT) [19], haptotherapy [20], biofeedback [21], enhanced antenatal care [22], muscle relaxation [23], yoga [24] etc. Different systematic review (SRs)/meta-analysis studies have been carried out to examine the effect of various interventions on reducing FoC. Abdolalipour et al. examined the effect of mindfulness-based interventions on FoC in a SR and meta-analysis. In this study, 5 trials were included in the meta-analysis and the evidence quality was moderate. The study concluded that these interventions probably reduce FoC [25]. In a SR and meta-analysis, Akgün et al. examined the effect of psychoeducation on reducing FoC. In this study, six heterogeneous trials were included in the meta-analysis. The findings proved psychoeducation to be effective in reducing FoC [6]. Alizadeh-Dibazari et al. examined the effect of prenatal education on reducing FoC in a SR and meta-analysis. In this study, 11 trials were included in the meta-analysis, the certainty of the evidence was low, and the findings indicated that prenatal education may reduce FoC [26]. Fathi Najafi et al. to examine the effect of CBT on reducing tocophobia, conducted a SR and meta-analysis including nine trials that were at a high level of heterogeneity. The results indicated that both internet-based CBT and traditional CBT are effective in reducing tocophobia [10]. In a SR and meta-analysis, Moghaddam Hosseini et al. examined the effect of various interventions on reducing FoC. Eight heterogeneous trials were included in the meta-analysis to examine the effect of educational interventions. The result showed that educational interventions were associated with a threefold reduction of FoC. In the subgroup analysis according to the type of educational interventions, the results showed that the effect of class education was significant for reducing FoC, yet the effect of psycho-education was insignificant. In this study, two homogenous trials were included in the meta-analysis to examine the effect of hypnosis interventions. The results showed that hypnosis interventions are associated with a 1.5-fold decrease FoC chance [12].

Considering the several SRs/meta-analyses conducted in regarding the effect of different non-pharmacological interventions on reducing FoC and the lack of an overview study in this field, we decided to comprehensively summarize relevant evidence from SRs published from trials to provide optimal evidence on the effect of different non-pharmacological interventions on reducing FoC and act in the clinic according to this evidence.

### Aims

The study was carried out to determine the effect of prenatal non-pharmacological interventions on reducing the FoC.

## Methods

The protocol for this overview has been published on PROSPERO (ID: CRD42023468547)

### Inclusion and exclusion criteria

#### Types of reviews

SR/meta-analysis studies carried out on RCTs or quasi-experimental studies examining the effect of prenatal non-pharmacological interventions on reducing FoC published in English or Farsi entered study. Other reviews and SRs on non-trial studies were excluded.

The original trials included in the SRs were extracted and analysed in terms of inclusion and exclusion criteria in the study to better report the effect of various interventions on reducing the FoC. Trials not meeting the inclusion criteria or meeting the exclusion criteria were not included in the meta-analysis.

#### Types of participants

The participants were the women in the first, second or third trimester of pregnancy with a high FoC according to the scale used in the study with no history of mental disorders.

#### Types of interventions and controls

SR/meta-analysis studies examining the effect of prenatal non-pharmacological interventions on reducing FoC and had a control group with routine prenatal care were included in the study.

The trials with more than one intervention in intervention group and/ or another intervention other than routine care in the control group were excluded.

#### Types of outcomes

The expected outcome of the study is the FoC, measured by standard tools such as The Wijma delivery expectancy/experience questionnaire (W-DEQ version A), FoC scale, and delivery fear questionnaire, before and after the intervention in the prenatal stage. The trials that examined the FoC score in the postpartum stage were excluded from the study.

#### Search strategy

PubMed, Web of science, Scopus, Cochrane, SID (Scientific Information Database) and Google Scholar search engines were systematically searched until July 27, 2023, with no time limits but limited to the studies published in English and Persian languages using the following keywords:

(“FoC” OR “fear of delivery” OR “childbirth related fear” OR “prenatal FoC” OR tokophobia OR tokophobia OR “expectation of childbirth” OR “experience of childbirth”) combined with (intervention OR *therapy OR counselling OR Psych* OR approach*) combined with ((“systematic review” OR “Systematic Review” OR “meta-analysis” OR “Meta Analysis” OR “Meta-analysis”).

The search strategies of various databases are seen in Appendix 1. In addition to the systematic search, a manual search was carried out in the references of the papers.

#### Study selection and data extraction

Two authors (Z A-D, MMa) independently reviewed the SRs in terms of inclusion and exclusion criteria. Thus, first the titles and then the abstract of the studies were examined, and if relevant, the full text of the studies was reviewed as well. Data extraction was carried out by two authors (Z, A-D, MMa) independently using a form designed for this beforehand. In cases with no agreement between the two authors (Z, A-D, MMa), it was resolved through consultation with the third author (MMi). Data extraction form included the first author`s name and the study publication date, the number of included trials to overview / the number of total trials of SR, the characteristics of the participants, the quality evaluation method of the included trials, the type of intervention group and control group, the evaluated outcomes, the conclusion, and whether or not the meta-analysis was performed.

#### Quality assessment

The Assessment of Multiple Systematic Reviews 2 (AMSTAR 2) checklist was used as a reliable and valid tool to assess the methodological quality of SRs and meta-analysis. It has 16 items, and the overall confidence for each item is scored as high, moderate, low, or critical. When a study has one or no non-critical weaknesses, it is considered as a high-quality study. Studies with more than one non-critical weaknesses are considered moderate-quality. Studies with one critical flaw with or without non-critical weaknesses are considered low-quality, and the ones with more than one critical flaw with or without non-critical weaknesses are seen as critically low-quality [27].

Two authors (Z, A-D, MMa) independently used Preferred Reporting Items for Systematic Reviews and Meta-Analyses (PRISMA) to assess SRs reporting quality. The checklist has 27 items. “Yes” or “No” were two possible responses for each item given based on each response [28].

Examining the certainty of evidence for interventions overall and separately was carried out by two authors (Z A-D, MMa) independently and any disagreements were resolved with a third author (MMi). The certainty of evidence was assessed using the grading system of recommendations, assessment development, and evaluation (GRADE) in five dimensions Risk of bias, Inconsistency, Indirectness, Imprecision and Publication bias [29]. If needed, original papers were reviewed too. In inconsistency assessment, all the trials included in the study were described and compared in terms of the characteristics of the studied population as well as the characteristics of the interventions provided to the study groups to examine the existence of clinical heterogeneity. I^2^ statistic and chi^2^ tests were used to examine the existence of statistical heterogeneity. In cases where I^2^ ≥50% or the chi^2^ test had a *p*-value less than 0.05, the certainty of the evidence was reduced because of inconsistency [30]. In indirectness evaluation, the study population, the type of intervention group and control group, and the outcomes of the studies were examined for answering the question of the current review [31]. In the evaluation of imprecision, the included trials were examined in terms of the enough participants to calculate the effect estimate (sample size > 400) and the size of the confidence interval around the effect estimate [32]. The quality of evidence was reduced by one degree if there is severe concern in any of the aspects, and two degrees in case of very severe concerns to calculate the quality of evidence for each of the examined outcomes.

#### Data synthesis

In order to better report the effect of different interventions on reducing the FoC, the data from the original trials entered in to SRs, were extracted and reanalysed in RevMan 5.3 using random-effect and in terms of standard mean difference (SMD) and 95% confidence interval (95% CI). Firstly, the overall effect of prenatal non-pharmacological interventions was analyzed on the outcome of FoC. Then the effect of each type of intervention (Psychological interventions, Prenatal educations, Distraction techniques and Enhanced cares) was analyzed on the outcome of FoC. Psychological interventions were mindfulness-based interventions, cognitive-behavioral therapy, psychoeducation and counseling; prenatal educations included training during pregnancy to prepare for childbirth; distraction techniques were relaxation, guided imagery, haptotherapy, biofeedback and yoga; and enhanced cares encompassed continuity cares, combination of one-to-one and group antenatal cares and companion-integrated childbirth preparation. Subgroup analysis was performed according to the type of study (RCTs and quasi-experimental). Subgroup analysis was not performed in the enhanced care interventions because all included studies were quasi-experimental. In psychological interventions, subgroup analysis was also performed according to the type of interventions (Mindfulness-based interventions, cognitive-behavioral therapy, psychoeducation, and counseling). The significance level was considered as *p*<0.05. To check the impact of high risk of bias studies on the general conclusion, sensitivity analysis was performed by removing high risk of bias studies. In studies with high heterogeneity, in addition to subgroup analysis, meta-regression models were also performed to evaluate the role of key variables such as the mean age of the mother, the sample size in the trials, the number of sessions and the duration of interventions in potential heterogeneity [33]. The impact of publication bias was assessed using Egger's test with a significance level of less than 0.05. [34] Comprehensive Meta Analysis V3 software was used to perform meta-regression models and Egger's test. Narrative synthesis was also performed for the results reported in SRs and their characteristics were presented in tables.

## Results

### Results of the literature search and study selection

**Fig. 1:**
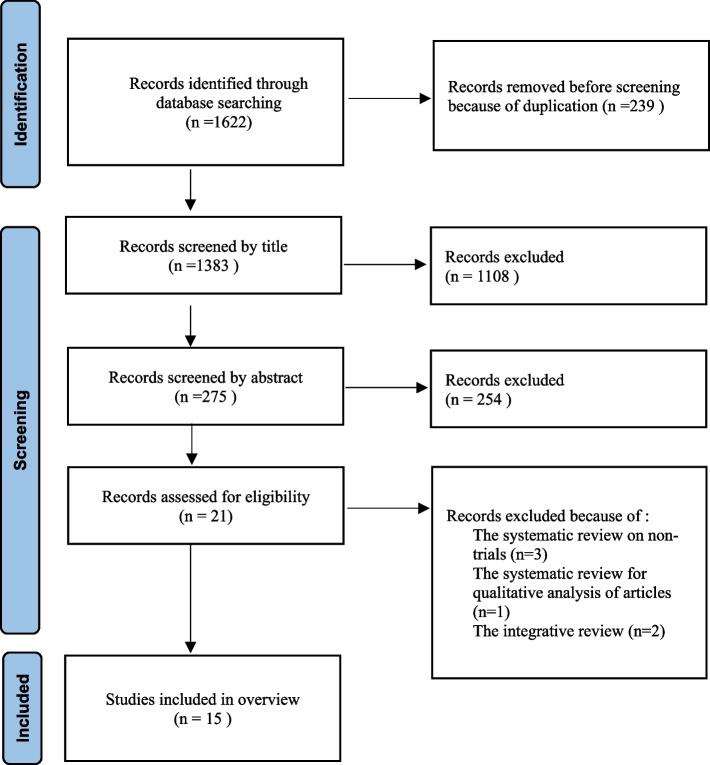
Flow diagram of the systematic literature search

Overall, 1622 studies were extracted from various databases and entered into EndNote 20. Of these, 239 studies were excluded because of duplication. A screen was carried out on 1383 studies, of which 1108 were excluded during the title screen and 254 during the abstract screen, then the full texts of the remaining 21 studies were examined, and three SRs [35–37] were excluded because of the study being carried out on studies other than trials. One SR [38] was excluded because of the qualitative analysis of the studies and two SRs [39, 40] because of conducting a study on quantitative, qualitative and mixed method studies, and 15 SRs were included in the study (Fig. 1).

### Characteristics of included SRs

The SRs in the study between 2018 and 2023 in the countries of Iran [8–10, 12, 25, 26, 41], Spain [42], Turkey [6], Australia [43], Singapore [33], United Kingdom [44, 45], Canada [46] and Nigeria [47] have been carried out. Among these SRs, the study of Azizi et al. [8] has been conducted on the trials carried out in Iran and the study of Tola et al. [47] has been conducted on trials conducted in low- and middle-income countries. The number of trials included in these SRs varied from seven [6, 44, 46] to 63 [9] and the number of participants from 728 [25] to 11,185 [9]. The number of authors in three SRs [6, 12, 26] is three, in four SRs [8, 25, 33, 42] four, in four SRs [41, 44–46] five, and in the other four SRs is six [9], seven [47], eight [10], and nine [43]. The interventions used in these SRs were psychological interventions [6, 8–10, 25, 33, 41, 42, 44–47], prenatal educations [8, 9, 12, 26, 42, 44, 45, 46], distraction techniques [8, 9, 12, 42, 45, 46] and enhanced cares [42, 43, 45, 46]. Besides FoC, other outcomes considered in SRs were self-efficacy [25, 47], birth type [6, 44], anxiety and depression among the pregnant women [33, 43, 44, 46, 47], birth preferences [44, 47], pain intensity during labor [26], epidural anesthesia during labor [44], childbirth experience, maternal attachment, and postpartum depression and anxiety [26]. All 15 studies included were SRs, and meta-analysis was carried out on 9 studies [6, 10, 12, 25, 26, 33, 41, 44, 45] (Table 1).

**Table 1:** Study characteristics of included systematic reviews

Authors/ Year	Included trials to overview/ total trials	Participants	Quality assessment	Interventions	Controls	Outcomes	Conclusion	Meta-analysis	PRISMA^a^ score
		Condition							
Abdolalipour et al. (2023) [25]	2/10	Pregnant women of any gestational age	The Cochrane handbook tool	Various mindfulness exercises: 1. MBSR^b^ 2. MBCP^c^ 3. MBCT^d^ 4. MBCE^e^ 5. Mindful Motherhood (MindBabyBody)	Routine prenatal care or training except mindfulness	FoC^f^ Self-efficacy	Mindfulness-based interventions probably reduce FOC and may promote self-efficacy	Yes	88.1
Adeli Gargari1 et al. (2021) [21]	7/9	Not reported	The Cochrane handbook tool	Effective theories, models, and interventions in the reduction of childbirth fear: 1. Prenatal education 2. Delivery counseling 3. Yoga and relaxation 4. Psychotherapy	Not reported	FoC Anxiety of childbirth	Psychologically-based interventions, can creatively play an essential role in reducing the FoC during pregnancy and even childbirth.	Yes	35.7
Aguilera-Martín et al. (2020) [42]	2/18	Pregnant women, with a low‐risk pregnancy and without mental disorders.	The Cochrane handbook tool	Nonmedical interventions, without pharmacological or surgical effect on participants: 1. Pregnancy‐related or birth‐related education 2. Psychological interventions 3. Alternative therapies 4. Continuous support	Usual care or absence of any intervention	Primary outcome: Fear of childbirth Secondary outcomes: 1. Pregnancy‐related or birth‐related anxiety 2. Fear of labor pain 3. Birth‐related self‐efficacy and coping ability 4. sense of control and safety during birth, 5. Catastrophization of birth.	Prenatal education, psychoeducation, and counseling proved to be effective to fight FoC and PrA^g^. Findings regarding relaxation techniques were contradictory and other therapies such as self‐hypnosis, mindfulness, art therapy, and MiCBT^h^, as well as physiotherapy interventions during labor, require more research.	No	78.6
Akgün et al. (2020) [6]	2/7	Women between the ages of 18 and 45 who were determined to have FoC by any measurement tool	Cochrane’s Risk of Bias instrument	Routine prenatal care with psychoeducation programs performed by health care providers in group or individual format through (a) internet-based, (b) computer-aided, (c)face-to-face or (d) telephone	Routine prenatal care	Primary outcome: Women’s FoC Secondary Outcome: CS^i^ rate	The positive effect of pure psychoeducation in decreasing the FoC was presented with high quality evidence in this systematic review and meta-analysis study. Psychoeducation is an effective method of decreasing the CS rate	Yes	71.4
Alizadeh-Dibazari et al. (2023) [26]	11/18	Pregnant women desiring a normal vaginal delivery and having no maternal or fetal problems history.	The Cochrane handbook tool	Structured prenatal education and routine prenatal care	Routine prenatal care	Primary outcomes: 1. Fear of childbirth 2. Pain intensity in the first and second labour phases 3. Childbirth experience Secondary outcomes 1. Maternal attachment 2. Postpartum depression 3. Postpartum anxiety	Providing prenatal education and routine care compared to providing only routine care may essentially decrease the fear of childbirth.	Yes	80.1
Azizi et al. (2021) [8]	17/21	Healthy Iranian pregnant women at each gestational age without psychological problems.	The modified Jadad Scale	Any type of interventions for moderating levels of FoC among pregnant women: 1. CBT^j^ 2. Relaxation techniques 3. CPCs^k^ 4. Psychological counseling 5. Mindfulness program	Not reported	FoC	Different interventions had been used for reducing FoC among pregnant women and most of them had shown effective results in this respect. There was no clear evidence to show the most effective method for decreasing levels of FoC among pregnant women.	No	75
Bakhteh et al.(2022) [9]	30/63	Not reported	CONSORT^l^ checklist	Treatment methods on reducing the fear or tokophobia of childbirth	Not reported	FoC	Psychotherapy and educational interventions decreased FoC. Education before childbirth, psychological education, cognitive- behavioral treatment, and consultation can alleviate FoC	No	64.3
Cibralic et al. (2023) [23]	1/8	Pregnant women over the age of 18 years	The mixed methods appraisal tool (50)	Midwifery-led continuity of care (MCoC)	Usual cares	Maternal mental health during the perinatal period: 1. Depression 2. Anxiety 3. Fear of birth	No significant differences found in fear of birth between women who received MCoC and those who did not	No	75
Fathi Najafi et al.(2021) [10]	5/9	Not reported	The Joanna Briggs Institute critical appraisal checklists.	Internet based CBT Traditional CBT	Other treatments	Tocophobia	CBT interventions significantly reduced tocophobia.	Yes	66.7
MoghaddaHosseini et al. (2017) [12]	5/10	The healthy pregnant (primiparous or multiparous) and postpartum women without restriction of language and time without major mental disorder regardless of age, type of birth and number of pregnancies	The Cochrane handbook tool	Any type of intervention such as: 1. Prenatal class education 2. Psycho-education 3. Consultation, 4. Supportive care 5. Different kinds of relaxation and 6. Relief pain techniques during labour	Prenatal and/or postnatal routine care	FoC during pregnancy and postpartum	Educational interventions were associated with about a three-fold reduction in the FOC. Hypnosis is associated with 1.5 time reduction in the chance of FOC.	Yes	73.8
Neo et al. (2022) [33]	1/15	pregnant women aged 18 years above during the first, second, and/or third trimesters.	The Cochrane Risk of Bias tool version 1	Any psychological principles such as: 1. Cognitive behavioral therapy 2. Mindfulness therapy 3. Problem solving therapy 4. Positive psychology 5. Psychoeducation 6. Psychodynamic psychotherapy 7. Acceptance commitment therapy 8. Interpersonal therapy 9.combination of these.	1. Treatment as usual 2. Waitlist control 3. Placebo control	Primary outcomes: 1. Depressive 2. Anxiety symptoms 3. Fear of childbirth Secondary outcome: Stress symptoms	Caution is needed in the interpretation of findings because of the few trials.	Yes	85.7
O'Connell et al.(2021) [44]	3/7	Women with high or severe FoC in pregnancy, as defined in each individual trial.	The Cochrane Handbook for Systematic Reviews of Interventions	Any non-pharmacological antenatal intervention aimed at reducing high to severe levels of FoC in women consisted of: 1. Psychosocial and psychological interventions 2. Physical exercise interventions 3. Therapeutic interventions	Standard or usual maternity care	Primary outcomes: Fear of childbirth Secondary outcomes: 1. Number of women having a caesarean section 2.Anxiety 3. Depression 4. Birth preferences 5. Epidural analgesia during labour.	Based on a small number of RCTs, the effects of non-pharmacological interventions for women with high to severe fear of childbirth are uncertain.	Yes	85.7
Stoll et al. (2018) [46]	4/7	Women with elevated childbirth fear, distress, trait-state anxiety, or elevated depression or without minimum threshold for PSA^m^/FoB,	Effective Public Health Practice Project Quality Assessment Tool	An intervention, educational component, or treatment regime for PSA/FoB: 1. A group prenatal care program 2. Intensive therapy for childbirth fear 3. Extended childbirth education 4. Antenatal yoga 5. Telephone psycho-education counselling	Not reported	1. Pregnancy specific Anxiety 2. Fear of childbirth	Short, individual psychotherapeutic interventions for childbirth fear and PSA delivered by maternity care providers with training in cognitive-behavioral therapy/psychotherapy are effective for women with elevated PSA/FoB. Interventions that were effective for pregnant women with a range of different PSA/FoB levels were childbirth education at the hospital (2 h), prenatal Hatha yoga (8 weeks), and an 8-week prenatal education course (16 h)	No	64.3
Tola et al. (2022) [47]	3/16	First-time mothers in low- and middle-income countries, with singleton pregnancies attending antenatal clinics, aged ≥18 years, without existing mental health diagnoses, without medical or obstetrics risks, and not planning to undergo an elective CD^n^	the Joanna Briggs Institute critical appraisal tool	Psychological, educational, or a combination of psychological and educational interventions	Usual care or other forms of nonpharmacologic interventions	1. Anxiety 2. Depression 3. Self-efficacy 4. knowledge decision-making about the birth method before birth and/or after birth	All psychoeducational intervention modes, such as antenatal education, antenatal counseling, antenatal training, and role play, had a significant effect on some of the psychological outcomes assessed, including childbirth attitude, fear of childbirth, depression, fear, and anxiety.	No	78.6
Webb et al. (2021) [45]	17/28	Women in the perinatal period with FoC or tokophobia	The Cochrane Risk of Bias Tool	Any intervention that was for women with FoC: 1. Cognitive behavioural therapy 2. Other talking therapies 3. Antenatal education 4. Enhanced midwifery care 5. Alternative interventions 6. Interventions during labour	Not reported	1. Fear of childbirth 2. Caearean section by choice	The most intervention approaches investigated reduce FOC.	Yes	81.1

^a^Preferred Reporting Items for Systematic Reviews and Meta-Analys; ^b^Mindfulness-Based Stress Reduction; ^c^ Mindfulness-Based Childbirth, and Parenting; ^d^Mindfulness-Based Cognitive Therapy; ^e^Mindfulness-Based Childbirth Education; ^f^Fear of Childbirth; ^g^Pregnancy‐related Anxiety; ^h^Mindfulness‐integrated Cognitive-Behavioral Therapy; ^i^Cesarean Section; ^j^Cognitive-Behavioral Therapy; ^k^Childbirth Preparation Classes; ^l^Consolidated Standards of Reporting Trials; ^m^Pregnancy-Specific Anxiety; ^n^Cesarean Delivery

Ninety trials were extracted from the 15 SRs included in the overview, of which 42 trials were excluded the meta-analysis because of lacking inclusion criteria or having the exclusion criteria, and meta-analysis was carried on using 48 trials. Table 1 shows the number of trials included in the meta-analysis from each SR per the total number of trials in each SR.

### Quality assessment of the SRs

AMSTAR 2 was used to assess the methodological quality of SRs. Of the 15 SRs in the study, seven SRS have a critical flaw with or without non-critical weaknesses, and were considered low quality [12, 26, 33, 42, 43, 44, 45], and 8 SRs have more than one critical flaw with or without non-critical weaknesses and were considered critically low-quality [6, 8–10, 25, 41, 46, 47]. Of the 16 items examined in the AMSTAR 2 tool, all studies were Yes or Partial Yes in terms of using a comprehensive resource search strategy, except for two studies [41, 47]. In all studies, the selection of the included studies had been carried out by two people independently. Excluding two studies [10, 41], the rest were Yes or Partial Yes in terms of using a satisfactory technique to examine the risk of bias. These studies had used various tools to evaluate the risk of bias like the Cochrane handbook tool [6, 12, 25, 26, 33, 42, 44, 45], the modified Jadad Scale [8], CONSORT checklist [9], the mixed methods appraisal tool [43], effective public health practice project quality assessment tool [46] and the Joanna Briggs Institute critical appraisal tool [47]. Except for two studies [12, 45], the rest had reported potential sources of conflict of interest and funding, and except four studies [12, 25, 26, 44], none had listed the excluded studies and the reason for their exclusion. Table 2 displays other characteristics of AMSTAR 2 scoring for SRs.

**Table 2:** Quality assessment of included reviews using the Assessment of Multiple Systematic Reviews 2 (AMSTAR 2)

Authors/ Year	AMSTAR 2 Items																Review’s quality
	1	2	3	4	5	6	7	8	9	10	11	12	13	14	15	16	
Abdolalipour et al. (2023) [25]	Y	Y	Y	PY	Y	Y	Y	PY	Y	N	N	N	Y	N	N	Y	Critically low
Adeli Gargari et al. (2021) [21]	N	N	Y	PY	N	N	N	N	Y	N	N	N	N	N	N	Y	Critically low
Aguilera-Martín et al. (2021) [42]	Y	Y	Y	Y	Y	Y	N	PY	Y	N	NMA	NMA	Y	Y	NMA	Y	Low
Akgün et al. (2020) [6]	Y	N	Y	PY	Y	Y	N	PY	Y	N	Y	Y	Y	Y	Y	Y	Critically low
Alizadeh-Dibazari et al. (2023) [26]	Y	Y	Y	PY	Y	Y	Y	PY	Y	N	N	N	Y	N	Y	Y	Low
Azizi et al. (2021) [8]	Y	N	Y	PY	Y	Y	N	PY	PY	N	NMA	NMA	Y	N	NMA	Y	Critically low
Bakhteh et al. (2022) [9]	N	N	N	PY	Y	Y	N	N	PY	N	NMA	NMA	N	N	NMA	Y	Critically low
Cibralic et al. (2023) [23]	Y	Y	N	PY	Y	Y	N	Y	PY	N	NMA	NMA	Y	N	NMA	Y	Low
Fathi Najafi et al. (2021) [10]	Y	N	Y	PY	Y	N	N	N	PY	N	Y	Y	Y	Y	Y	Y	Critically low
MoghaddamHosseini et al. (2018) [12]	Y	N	Y	Y	Y	N	Y	N	Y	N	Y	N	Y	Y	Y	N	Low
Neo et al. (2022) [33]	Y	Y	Y	PY	Y	Y	N	Y	Y	Y	Y	N	Y	Y	Y	Y	Low
O'Connell et al. (2021) [44]	Y	Y	Y	Y	Y	Y	Y	Y	Y	Y	Y	Y	Y	Y	N	Y	Low
Stoll et al. (2018) [46]	N	N	N	PY	Y	N	N	PY	PY	N	NMA	NMA	N	N	NMA	Y	Critically low
Tola et al. (2022) [47]	Y	N	Y	PY	N	Y	N	Y	PY	N	NMA	NMA	Y	Y	NMA	N	Critically low
Webb et al. (2021) [45]	Y	Y	N	PY	Y	Y	N	N	Y	N	Y	N	Y	Y	Y	Y	Low

Highlighted columns are AMSTAR 2 critical domains

Y: Yes, PY: Partial Yes, N: No, NMA: No meta-analysis was conducted

Low: One critical flaw with or without non-critical weaknesses: the review has a critical flaw and may not provide an accurate and comprehensive summary of the available studies that address the question of interest

Critically low: More than one critical flaw with or without non-critical weaknesses: the review has more than one critical flaw and should not be relied on to provide an accurate and comprehensive summary of the available studies

1. Did the research questions and inclusion criteria for the review include the components of PICO?

2. Did the report of the review contain an explicit statement that the review methods were established prior to the conduct of the review and did the report justify any significant deviations from the protocol?

3. Did the review authors explain their selection of the study designs for inclusion in the review?

4. Did the review authors use a comprehensive literature search strategy?

5. Did the review authors perform study selection in duplicate?

6. Did the review authors perform data extraction in duplicate?

7. Did the review authors provide a list of excluded studies and justify the exclusions?

8. Did the review authors describe the included studies in adequate detail?

9. Did the review authors use a satisfactory technique for assessing the risk of bias (RoB) in individual studies that were included in the review?

10. Did the review authors report on the sources of funding for the studies included in the review?

11. If meta-analysis was performed did the review authors use appropriate methods for statistical combination of results?

12. If meta-analysis was performed, did the review authors assess the potential impact of RoB in individual studies on the results of the meta-analysis or other evidence synthesis?

13. Did the review authors account for RoB in individual studies when interpreting/discussing the results of the review?

14. Did the review authors provide a satisfactory explanation for, and discussion of, any heterogeneity observed in the results of the review?

15. If they performed quantitative synthesis did the review authors carry out an adequate investigation of publication bias (small study bias) and discuss its likely impact on the results of the review?

16. Did the review authors report any potential sources of conflict of interest, including any funding they received for conducting the review?

Evaluation of SRs reporting quality using PRISMA revealed that almost all SRs with meta-analysis except two studies [10, 41], and almost all SRs without meta-analysis except two studies [9, 46] were over 70 percent consistent with PRISMA checklist, showing a relatively complete report. The details of this evaluation are given in Table 3.

**Table 3:** Reporting quality of the included systematic reviews

**Section/topic**		Abdolalipour et al. (2023) [25]	Adeli Gargari et al. (2021) [21]	Aguilera-Martín et al. (2021) [42]	Akgün et al. (2020) [6]	Alizadeh-Dibazari et al. (2023) [26]	Azizi et al. (2021) [8]	Bakhteh et al. (2022) [9]	Cibralic et al. (2023) [23]	Fathi Najafi et al. (2021) [10]	MoghaddamHosseini et al. (2018) [12]	Neo et al. (2022) [33]	O'Connell et al. (2021) [44]	Stoll et al. (2018) [46]	Tola et al. (2022) [47]	Webb et al. (2021) [45]
**Title**	Title^1^	Y	Y	Y	Y	Y	Y	Y	Y	Y	Y	Y	Y	Y	Y	Y
**Abstract**	Abstract^2^	Y	N	N	N	N	N	N	N	N	N	Y	N	N	Y	N
**Introduction**	Rationale^3^	Y	Y	Y	Y	Y	Y	Y	Y	Y	Y	Y	Y	Y	Y	Y
	Objectives^4^	Y	N	Y	N	Y	N	Y	Y	Y	Y	Y	Y	Y	Y	Y
**Methods**	Eligibility criteria^5^	Y	N	Y	Y	Y	Y	Y	Y	Y	Y	Y	Y	Y	Y	Y
	Information sources^6^	Y	Y	Y	Y	Y	Y	Y	Y	Y	Y	Y	Y	Y	Y	Y
	Search strategy^7^	Y	N	Y	N	Y	Y	N	Y	N	N	N	N	N	N	Y
	Selection process^8^	Y	Y	Y	Y	Y	Y	Y	N	Y	Y	Y	Y	Y	Y	Y
	Data collection process^9^	Y	N	Y	Y	Y	Y	Y	N	N	Y	Y	Y	N	Y	Y
	Data items^10a^	Y	Y	Y	Y	Y	Y	Y	Y	Y	Y	Y	Y	Y	Y	Y
	Data items^10b^	Y	N	Y	Y	Y	Y	Y	N	N	Y	Y	Y	N	Y	Y
	Study risk of bias assessment ^11^	Y	Y	Y	Y	Y	Y	Y	Y	Y	Y	Y	Y	Y	Y	Y
	Effect measures^12^	Y	N	N	Y	Y	Y	Y	N	N	Y	N	Y	N	Y	Y
	Synthesis methods^13a^	Y	N	NA	Y	Y	NA	NA	NA	Y	Y	Y	Y	NA	NA	Y
	Synthesis methods^13b^	Y	N	NA	Y	Y	NA	NA	NA	Y	Y	Y	Y	NA	NA	Y
	Synthesis methods^13c^	Y	N	NA	Y	Y	NA	NA	NA	Y	Y	Y	Y	NA	NA	Y
	Synthesis methods^13d^	Y	N	NA	Y	Y	NA	NA	NA	Y	Y	Y	Y	NA	NA	Y
	Synthesis methods^13e^	Y	N	NA	Y	N	NA	NA	NA	Y	Y	Y	Y	NA	NA	Y
	Synthesis methods^13f^	N	N	NA	Y	N	NA	NA	NA	Y	Y	Y	Y	NA	NA	Y
	Reporting bias assessment^14^	N	N	NA	N	N	NA	NA	NA	N	N	N	N	NA	NA	N
	Certainty assessment^15^	Y	N	NA	N	Y	NA	NA	NA	N	N	Y	Y	NA	NA	N
**Results**	Study selection^16a^	Y	Y	Y	Y	Y	Y	Y	Y	Y	Y	Y	Y	Y	Y	Y
	Study selection^16b^	Y	N	N	N	Y	N	N	N	N	Y	N	Y	N	N	N
	Study characteristics^17^	Y	Y	Y	Y	Y	Y	Y	Y	Y	Y	Y	Y	Y	Y	Y
	Risk of bias in studies^18^	Y	N	Y	Y	Y	Y	N	Y	Y	Y	Y	Y	Y	Y	Y
	Results of individual studies^19^	Y	Y	Y	Y	Y	Y	Y	Y	Y	Y	Y	Y	Y	Y	Y
	Results of syntheses ^20a^	Y	Y	NA	Y	Y	NA	NA	NA	Y	Y	Y	Y	NA	NA	Y
	Results of syntheses ^20b^	Y	Y	NA	Y	Y	NA	NA	NA	Y	Y	Y	Y	NA	NA	Y
	Results of syntheses ^20c^	Y	N	NA	Y	N	NA	NA	NA	Y	Y	Y	Y	NA	NA	Y
	Results of syntheses ^20d^	N	N	NA	Y	N	NA	NA	NA	Y	Y	Y	Y	NA	NA	Y
	Reporting biases^21^	N	N	NA	N	N	NA	NA	NA	N	N	N	N	NA	NA	N
	Certainty of evidence^22^	Y	N	NA	N	Y	NA	NA	NA	N	N	Y	Y	NA	NA	N
**Discussion**	Discussion^23a^	Y	Y	Y	Y	Y	Y	Y	Y	Y	Y	Y	Y	Y	Y	Y
	Discussion^23b^	Y	N	Y	Y	Y	Y	Y	Y	Y	Y	Y	Y	Y	Y	Y
	Discussion^23c^	Y	N	Y	Y	Y	Y	Y	Y	Y	Y	Y	Y	Y	Y	Y
	Discussion^23d^	Y	Y	Y	Y	Y	Y	Y	Y	Y	Y	Y	Y	Y	Y	Y
**Other information**	Registration and protocol^24a^	Y	N	Y	N	Y	N	N	Y	N	N	Y	Y	N	Y	Y
	Registration and protocol^24b^	Y	N	Y	N	Y	N	N	Y	N	N	Y	Y	N	Y	Y
	Registration and protocol^24c^	N	N	N	N	N	N	N	N	N	N	N	N	N	N	N
	Support^25^	Y	Y	N	Y	Y	Y	N	Y	Y	Y	Y	Y	Y	N	Y
	Competing interests^26^	Y	Y	Y	Y	Y	Y	Y	Y	Y	N	Y	Y	Y	N	Y
	Availability of data, code and other materials^27^	Y	N	N	N	Y	N	N	Y	N	N	Y	N	N	N	N
The percentage of Y*		88.1	35.7	78.6	71.4	80.1	75	64.3	75	66.7	73.8	85.7	85.7	64.3	78.6	81

Highlighted columns are systematic reviews without meta-analysis

Unhighlighted columns are systematic reviews and meta-analysis

*Y* Yes, *N* No, *NA* Not applicable

Checking the certainty of evidence was conducted for interventions overall and separately considering the assessment of the writers of the entered SRs. If not reported, the certainty of evidence was assessed by the authors of the present study. For non-pharmacological prenatal interventions, the confidence level of the evidence was considered very low. Regarding the risk of bias and publication bias, it was in a serious level, in terms of imprecision and indirectness in a no serious level, and in terms of inconsistency, it was in a very serious level.

The certainty of evidence for each intervention was evaluated as follows: in terms of risk of bias, except enhanced cares, which was in no serious level, the rest of the interventions were in serious level. In terms of inconsistency, the distraction techniques were in the no serious level and the rest of the interventions were in the serious level. Considering indirectness, all interventions were no serious. Given imprecision, enhanced cares were in a very serious level. Ultimately, in assessing of publication bias, psychological interventions and prenatal educations were in serious level. Overall, the certainty of evidence for distraction techniques was considered moderate and the rest of the interventions very low (Table 4).

**Table 4:** Quality assessment of included studies according to GRADE^a^ approach

**Risk of bias**	**Inconsistency**	**Indirectness**	**Imprecision**	**Publication bias**	**Pooled effect size** **(95% CI**^**b**^**)**	**Final judgment** **(Certainty)**
**Non-pharmacological prenatal interventions**						
Serious	Very Serious	No serious	No serious	Serious	SMD -1.32 (-1.60 to -1.03)	⊕⊖⊖⊖ Very Low
**Psychological interventions**						
Serious	Serious	No serious	No serious	Serious	SMD^c^ -1.56 (-2.01 to -1.11)	⊕⊖⊖⊖ Very Low
**Prenatal educations**						
Serious	Serious	No serious	No serious	Serious	SMD -1.18 (-1.83 to 0.52)	⊕⊖⊖⊖ Very Low
**Distraction techniques**						
Serious	No serious	No serious	No serious	No serious	SMD -0.75 (-0.98 to 0.51)	⊕⊕⊕⊖ Moderate
**Enhanced cares**						
No Serious	Serious	No serious	Very Serious	No serious	SMD -1.14 (-2.85 to 0.58)	⊕⊖⊖⊖ Very Low

^a^*GRADE* Grading of Recommendations Assessment, Development and Evaluation; ^b^*CI* confidence interval, ^c^*SMD* standardized mean difference

GRADE Working Group grades of evidence

High certainty: We are very confident that the true effect lies close to that of the effect estimate

Moderate certainty: We are moderately confident in the effect estimate; the true effect is likely to be close to the estimate of the effect, but there is a possibility that it is substantially different

Low certainty: Our confidence in the effect estimate is limited; the true effect may be substantially different from the estimate of the effect

Very low certainty: We have very little confidence in the effect estimate; the true effect is likely to be substantially different from the estimate of the effect

### The results of our meta-analysis

Of the 15 SRs included in the study, the data of 48 trials were extracted and meta-analysis was done based on the interventions as a whole and separately. Later on, sub-group analysis was carried out according to the type of study. Forty-two trials were excluded from the meta-analysis because of lack of inclusion criteria.

### Non-pharmacological prenatal interventions

The results of 48 studies (25 RCTs and 23 quasi-experimental studies) revealed that non-pharmacological prenatal interventions compared to prenatal usual care resulted in a significant reduction in FoC (SMD -1.32, 95% CI -1.60 to -1.03, 48 trials, 4871 participants, I^2^ = 95%).

Given the high heterogeneity, meta-regression models were carried out to assess the role of key variables such as the mean age of the mother, the sample size in the trial, the number of sessions and the duration of interventions on FoC. However, there were no significant relationships between the sample size in the trial, the number of sessions and the duration of interventions as the confounding factors on the FoC (*p*-values were respectively 0.20, 0.10, and 0.22). Nonetheless, there was a significant correlation between mother's age and FoC, as the mother's age increases, the mean score of FoC increases too (β=0.129, *P*=0.023) (Table 5). Egger's test findings revealed publication bias (*p*<0.001).

**Table 5:** Meta-regression analysis of variables predicting fear of childbirth

Variables	Number of studies	Regression coefficient (SE)	95% CI	*p* value	Q (model)
**Mean Age**					
Psychological interventions	28	0.128 (0.102)	-0.072 to 0.329	0.210	1.57
Prenatal educations	14	0.078 (0.137)	-0.192 to 0.348	0.571	0.32
Non-pharmacological prenatal interventions	51	0.129 (0.057)	0.017 to 0.241	0.023	5.16
**Total sample size**					
Psychological interventions	28	0.016 (0.012)	-0.008 to 0.041	0.202	1.63
Prenatal educations	14	0.0002 (0.021)	-0.041 to 0.041	0.991	0.00
Non-pharmacological prenatal interventions	52	0.010 (0.008)	-0.006 to 0.027	0.206	1.59
**Number of sessions**					
Psychological interventions	28	-0.070 (0.105)	-0.276 to 0.135	0.501	0.45
Prenatal educations	14	0.094 (0.097)	-0.09 to 0.028	0.335	0.93
Non-pharmacological prenatal interventions	51	0.069 (0.043)	-0.014 to 0.153	0.106	2.61
**Duration of psychological interventions**					
Psychological interventions	28	-0.0004 (0.001)	-0.002 to 0.001	0.730	0.12
Prenatal educations	14	0.0005 (0.0007)	-0.0009 to 0.002	0.487	0.48
Non-pharmacological prenatal interventions	51	0.0006 (0.0005)	-0.0004 to 0.001	0.229	1.45

### Psychological interventions

**Fig. 2:**
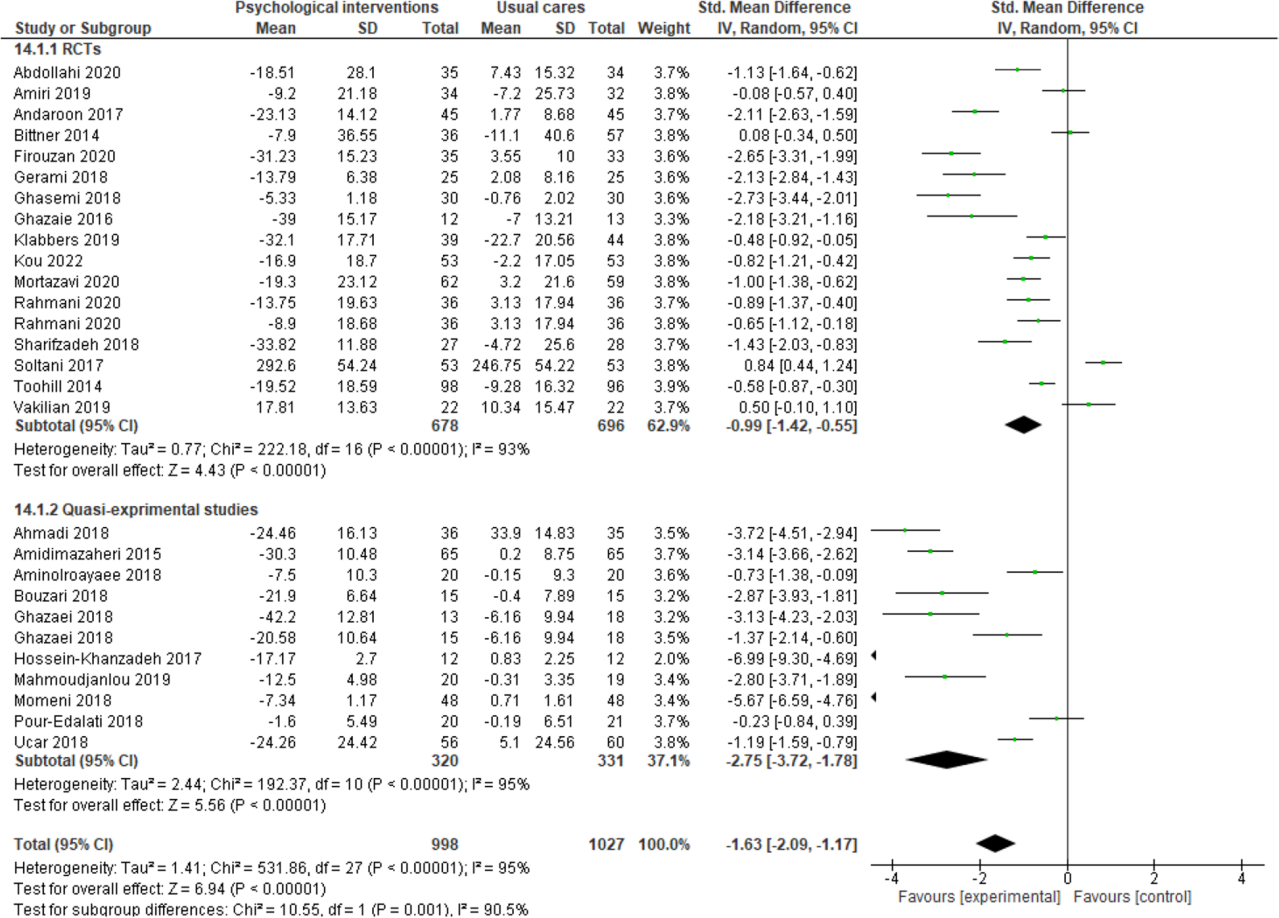
Psychological interventions versus routine prenatal cares, Sub-group analysis based on study design, Outcome: Fear of childbirth

The findings of 28 studies (17 RCTs and 9 quasi-experimental studies) indicated that psychological interventions compared to prenatal usual cares lead to a significant reduction in FoC (SMD -1.63, 95% CI -2.09 to -1.17, 28 trials, 2025 participants, I^2^ = 95%) (Fig. 2).

Sub-group analysis findings according to the type of study for RCTs (SMD -0.99, 95% CI -1.42 to -0.55, 17 trials, 1374 participants, I^2^ = 93%) and quasi-experimental studies (SMD -2.75, 95% CI -3.72 to -1.78, 9 trials, 651 participants, I^2^ = 95%) showed a significant decrease in FoC in the recipients of psychological interventions compared to the recipients of prenatal usual care (Fig. 2).

**Fig. 3:**
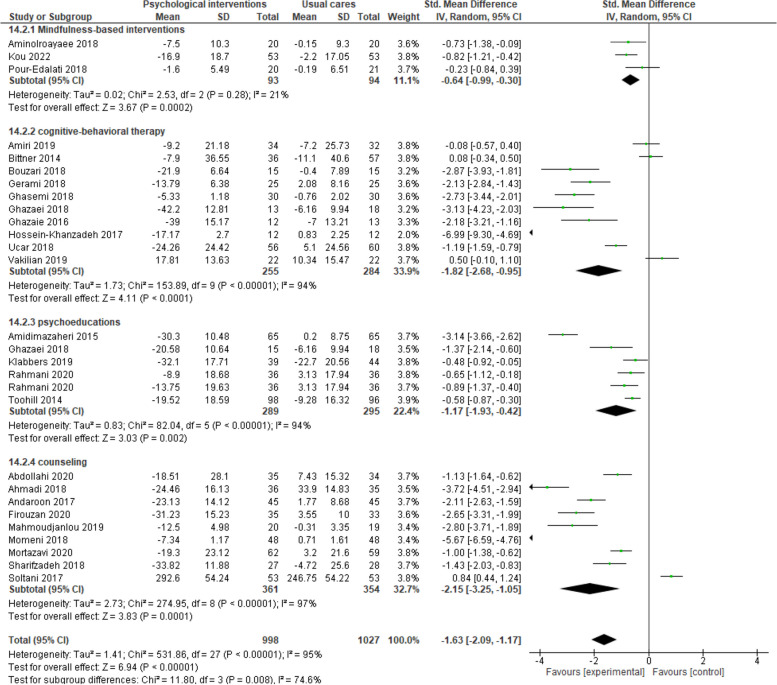
Psychological interventions versus routine prenatal cares, Sub-group analysis based on the kind of psychological interventions, Outcome: Fear of childbirth

The outcome of sub-group analysis according to the type of psychological interventions revealed that in the recipients of mindfulness-based interventions (SMD -0.64, 95% CI -0.99 to -0.30, 3 trials, 187 participants, I^2^ = 21%), cognitive-behavioral therapy (SMD -1.82, 95% CI -2.68 to -0.95, 10 trials, 539 participants, I^2^ = 94%), psychoeducation (SMD -1.17, 95% CI -1.93 to -0.42, 6 trials, 584 participants, I^2^ = 94%) and counseling (SMD -2.15, 95% CI -3.25 to -1.05, 9 trials, 715 participants, I^2^ = 97%) compared to recipients of prenatal usual care, there is a significant reduction in FoC (Fig. 3).

Sensitivity analysis was carried out by removing high risk of bias studies to examine the effect of high risk of bias studies on the general conclusion. The findings revealed that psychological interventions compared to prenatal usual cares cause a significant reduction in FoC (SMD -2.02, 95% CI -2.69 to -1.36, 16 trials, 1057 participants, I^2^ = 95%).

Given the high heterogeneity, besides subgroup analysis, meta-regression models were conducted to assess the role of key variables such as the mean age of the mother, the sample size in the trial, the number of sessions and the duration of psychological interventions on FoC; however, no significant relationships were reached (the *p*-values were respectively 0.21, 0.20, 0.50 and 0.73) (Table 5). Egger's test results indicate publication bias (*p*<0.001).

### Prenatal education

**Fig. 4:**
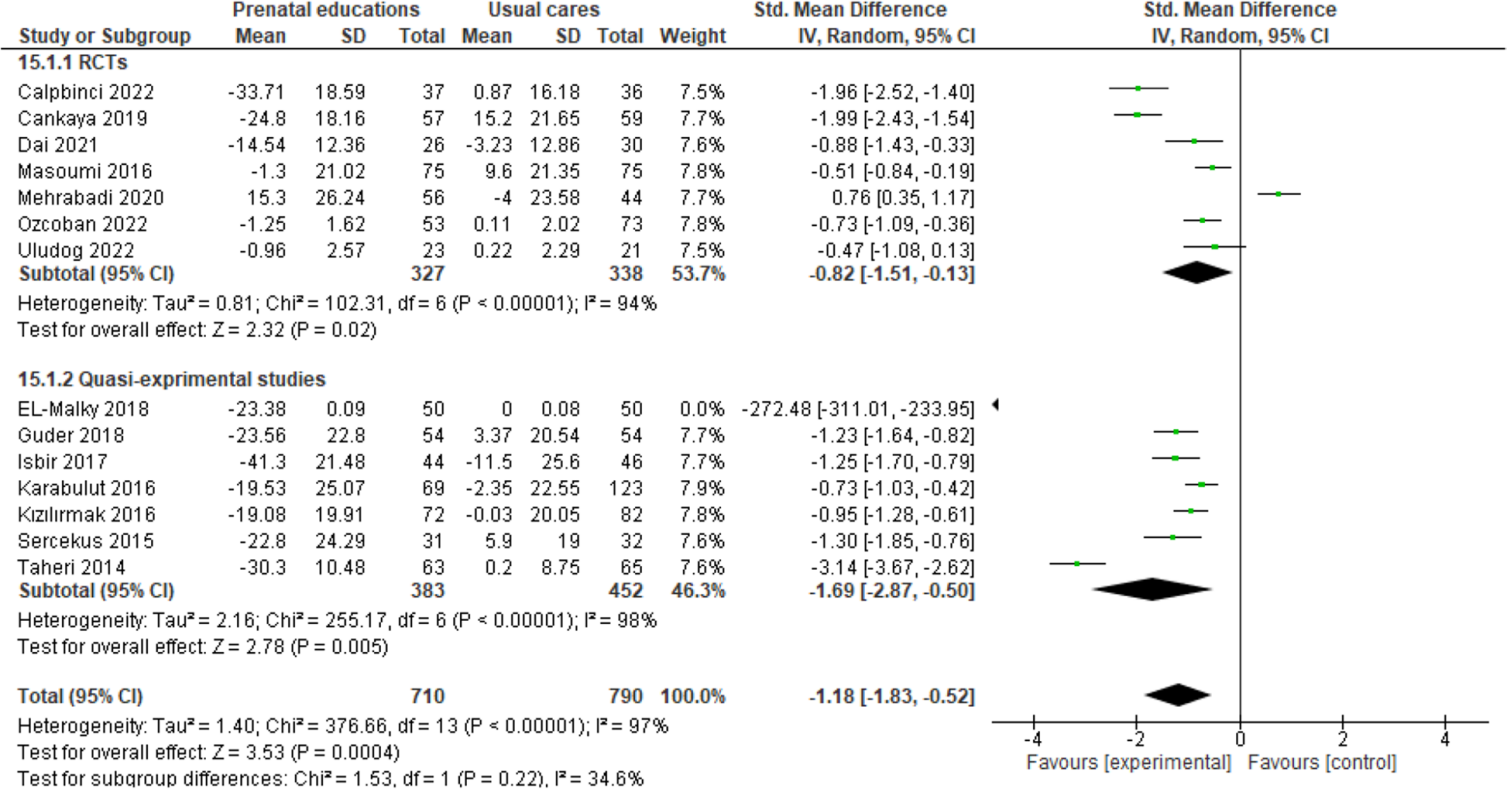
Prenatal educations versus routine prenatal cares, Sub-group analysis based on study design, Outcome: Fear of childbirth

The results of 14 studies (7 RCTs and 7 quasi-experimental studies) revealed that prenatal educations compared to prenatal usual cares lead to a significant reduction in FoC (SMD -1.18, 95% CI -1.83 to -0.52, 14 trials, 1500 participants, I^2^ = 97%) (Fig. 4).

The results of subgroup analysis according to the type of study for RCTs (SMD -0.82, 95% CI -1.51 to -0.13, 7 trials, 665 participants, I^2^ = 94%) and quasi-experimental studies (SMD -1.69, 95% CI -2.87 to -0.50, 7 trials, 835 participants, I^2^ = 98%) showed a significant reduction in FoC in prenatal education recipients compared to prenatal usual care recipients (Fig. 4).

Sensitivity analysis was carried out to remove high risk of bias studies and the results revealed that prenatal educations relative to prenatal usual cares cause a significant reduction in FoC (SMD -0.88, 95% CI -1.16 to -0.61, 4 trials, 432 participants, I^2^ = 72.8%).

In the meta-regression models, there were no significant relationships between key variables such as the mean age of the mother, the sample size in the trial, the number of sessions and the length of prenatal educations with the level of FoC (*p*-values were respectively, 0.57, 0.99, 0.33, and 0.48) (Table 5). The findings of Egger’s test revealed publication bias (*p*=0.002).

### Distraction techniques

**Fig. 5:**
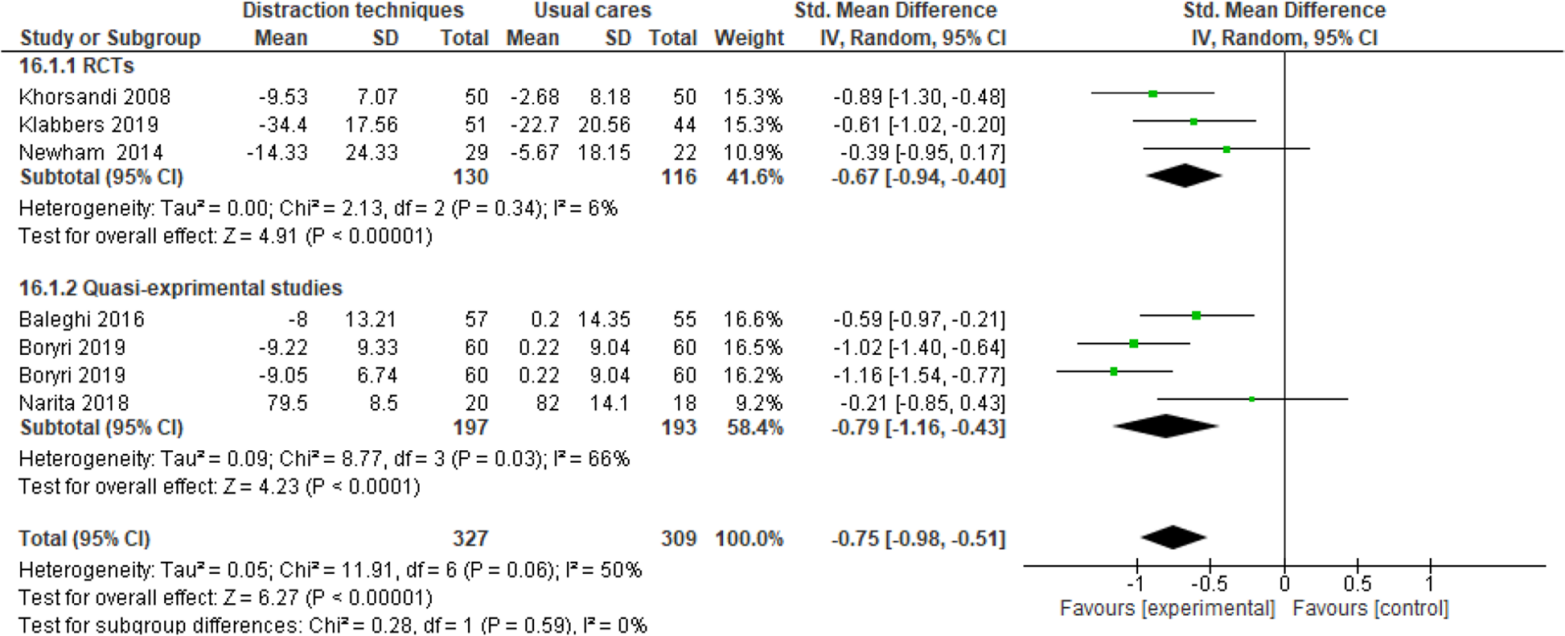
Distraction techniques versus routine prenatal cares, Sub-group analysis based on study design, Outcome: Fear of childbirth

The results of 7 studies (3 RCTs and 4 quasi-experimental studies) revealed that distraction techniques compared to prenatal usual cares result in a significant reduction in FoC (SMD -0.75, 95% CI -0.98 to -0.51, 7 trials, 636 participants, I^2^ = 50%) (Fig. 5).

Sub-group analysis results according to study type for RCTs (SMD -0.67, 95% CI -0.94 to -0.40, 3 trials, 246 participants, I^2^ = 60%) and quasi-experimental studies (SMD -0.79, 95% CI -1.16 to -0.43, 4 trials, 390 participants, I^2^ = 66%) indicated a significant decrease in FoC among the recipients of distraction techniques compared to the recipients of prenatal usual care (Fig. 5).

Sensitivity analysis was conducted to do away with high risk of bias studies where the findings showed that distraction techniques compared to prenatal usual cares cause a significant reduction in FoC (SMD -0.75, 95% CI -1.18 to -0.33, 4 trials, 329 participants, I^2^ = 69%). Egger’s test results indicate the absence of publication bias as well (*p*=0.07).

### Enhanced cares

**Fig. 6:**
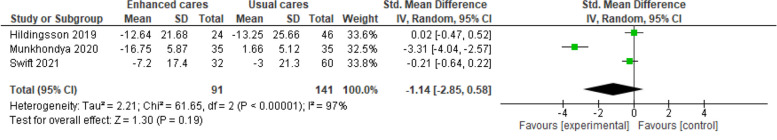
Enhanced cares versus routine prenatal cares, Outcome: Fear of childbirth

The results of 3 quasi-experimental studies showed that enhanced cares do not significantly reduce FoC compared to prenatal usual cares (SMD -1.14, 95% CI -2.85 to 0.58, 3 trials, 232 participants, I^2^ = 97%) (Fig. 6).

## Discussion

The study is the first overview to that comprehensively examined the effect of different non-pharmacological interventions on reducing FoC. To do so, 15 SRs with or without meta-analysis entered in the study, all of which were in low quality or critically low quality in terms of methodological quality, yet had relatively complete reports in terms of reporting quality.

The certainty of evidence regarding non-pharmacological prenatal interventions was evaluated as very low, which was in a very serious level in terms of inconsistency and in a serious level regarding the risk of bias and publication bias. The results of the meta-analysis showed that non-pharmacological prenatal interventions compared to prenatal usual care may decrease FoC in mothers, but the evidence is very uncertain.

Regarding psychological interventions, the certainty of evidence was examined as very low, which was in a serious level from publication bias, inconsistency and risk of bias perspectives. The results of the meta-analysis showed that psychological interventions compared to prenatal usual care may decrease FoC in mothers, but the evidence is very uncertain. Following the removal of high risk of bias studies in the sensitivity analysis, the level of certainty of evidence increased to a low level, with the results indicating that psychological interventions compared to prenatal usual care may decrease FoC in mothers. Further, the results of sub-group analysis revealed that all types of psychological interventions (Mindfulness-based interventions, cognitive-behavioral therapy, psychoeducation, and counseling) could decrease FoC compared to prenatal usual care, but the evidence is very uncertain.

Assessing the certainty of evidence regarding prenatal educations showed a very low level, where publication bias, inconsistency and risk of bias were in a serious situation too. Meta-analysis findings revealed that prenatal educations relative to usual prenatal cares may decrease FoC, but the evidence is very uncertain. The level of certainty of evidence increased to a low level following the removal of high risk of bias studies in the sensitivity analysis, and the results showed that prenatal education compared to prenatal usual care may decrease FoC in mothers.

In distraction techniques, examining certainty of evidence revealed a moderate level, which was in a serious level in terms of risk of bias. Meta-analysis results revealed that distraction techniques, compared to prenatal usual cares, probably decrease FoC. The level of certainty of evidence increased to a high level following the removal of high risk of bias studies in sensitivity analysis, and the results showed that distraction techniques decrease FoC in mothers compared to prenatal usual care.

The certainty of evidence in enhanced cares was evaluated as very low, which was very serious in terms of imprecision and serious in terms of inconsistency. The results of the meta-analysis showed that enhanced cares relative to prenatal usual cares may have no effect on reducing FoC, yet the evidence is very uncertain.

In a SR with meta-analysis, O'Connell et al. examined the effectiveness of non-pharmacological interventions compared to standard maternal care on reducing FoC in women with severe FoC. Seven trials with 1357 participants were included in the study. The interventions used in the studies were psychoeducation, cognitive behavioral therapy, group discussion, peer education, and art therapy. In this study, the certainty of evidence was reduced because of concerns about the risk of bias, imprecision and inconsistency. The results showed that using non-pharmacological interventions may decrease the level of FoC, but this decrease might not be clinically significant [44].

In a SR along with Meta-analysis, Moghaddam Hosseini et al. examined effective interventions in reducing FoC. Ten trails with 3984 participants were included in the study, in 8 studies the effect of training and in 2 studies the effect of hypnosis-based intervention on reducing FoC were examined. The results showed that both interventions lead to the reduction of FoC, but the effect of training on reducing FoC was twice that of the effect of hypnosis-based intervention [12].

A SR with meta-analysis was conducted by Webb et al. to identify effective interventions in reducing FoC including 28 studies. The interventions identified in the study are divided into six groups, including cognitive behavioral therapy, other talking therapies, antenatal education, enhanced midwifery care, alternative interventions and interventions during labor. The meta-analysis showed that most interventions regardless of the type of intervention reduce FoC, yet the poor methodological quality of the included studies leads to limited conclusions and quality RCTs are needed for future conclusions [45].

Akgün et al. studied the effect of psychoeducation on the reduction of FoC in a SR and meta-analysis. This SR had 4 RCTs, 3 non-randomized controlled studies and 931 participants, where psychoeducation was provided as a group or individually via internet-based, computer-aided, face-to-face or telephone. The results brought about enough evidence that psychoeducation is effective in reducing FoC [6].

Neo et al. examined the effects of internet-delivered psychological interventions on reducing symptoms of depression, anxiety and FoC in a SR and meta-analysis. In this SR, 16 RCTs including 3894 pregnant women from 23 countries were studied. Meta-analysis results revealed that internet-delivered psychological interventions bring about a significant reduction in depression and anxiety symptoms compared to usual care during pregnancy. However, there was insufficient evidence about its effect on reducing FoC and stress symptoms. Subgroup analyses indicated that the types of cognitive behavioral therapy or mindfulness therapy have beneficial effects in reducing depression symptoms among psychological interventions, yet the certainty of evidence for the outcomes of the study was low to very low [33].

### Strengths and limitations

Among the strengths of the study were registering the protocol of study in Prospero prior to the start of the study; examining certainty of evidence using the GRADE system, methodological quality using the AMSTAR 2 tool and reporting quality using the PRISMA Score; re-meta-analysis on the raw data extracted from original trials, performing sub-group analysis to examine the effect of study design on the study result, and conducting sensitivity analysis in order to eliminate the effects of studies with high risk of bias on the study outcomes.

Among the limitations were language limitations in entering SRs in Farsi and English, low to critically low methodological quality in SRs entered into the study, and very low to low level of evidence quality in some interventions to reduce FoC, all of which result in limitations in the study conclusion.

## Conclusion

The overview findings regarding SRs indicated that distraction techniques are effective in reducing FoC. Regarding the effect of psychological interventions and prenatal educations on FoC reduction, the findings indicted that these interventions may bring about a reduction of FoC; however, RCTs with high sample size and methodological quality are required for definitive conclusions. Concerning the effect of enhanced cares in reducing FoC, very uncertain evidence showed that these cares are ineffective in reducing FoC, and RCTs with high sample size and methodological quality are required to reach definite conclusions in this regard.

### Supplementary Information

Supplementary Material 1.

## Data Availability

The original contributions presented in the study are included in the article. Further inquiries can be directed to the corresponding author.

## Abbreviations

FoC: Fear of childbirth
SID: Scientific information database
SR: Systematic review
SMD: Standardized mean difference
CI: Confidence interval
PTSD: Post-traumatic stress disorder
VB: Vaginal birth
CBT: Cognitive-behavioral therapy
RCT: Randomized controlled trial
W-DEQ: Wijma delivery expectancy/experience questionnaire
AMSTAR 2: The Assessment of Multiple Systematic Reviews 2
PRISMA: Preferred Reporting Items for Systematic Reviews and Meta-Analyses
GRADE: The grading system of recommendations, assessment development, and evaluation
