# Supplementary material for: The effect of non-pharmacological prenatal interventions on fear of childbirth: an overview of systematic reviews and meta-analysis
Source: BMC Psychiatry. 2024 Jun 4;24:415. doi: 10.1186/s12888-024-05870-5 (PMC11151647; doi:10.1186/s12888-024-05870-5)
Supplement: Supplementary file 1 — Supplementary Material 1. [file 12888_2024_5870_MOESM1_ESM.docx]

**Appendix 1:** Search Strategy

**PubMed**

((((((((((''fear of childbirth''[Text Word]) OR (''tokophobia''[Text Word])) OR (''fear of delivery''[Text Word])) OR (''childbirth related fear''[Text Word])) OR (''expectation of childbirth''[Text Word])) OR (''experience of childbirth''[Text Word])) OR (''prenatal fear of childbirth''[Text Word])) OR (''postnatal fear of childbirth''[Text Word])) OR (tocophobia[Text Word])) AND (((((counselling[Text Word]) OR (intervention[Text Word])) OR (*therapy[Text Word])) OR (Psych*[Text Word])) OR (approache*[Text Word]))) AND (((review[Text Word]) OR (''systematic review''[Text Word])) OR (meta-analysis[Text Word]))

**Scopus**

( ( TITLE-ABS-KEY ( ''fear AND of AND childbirth'' ) OR TITLE-ABS-KEY ( ''fear AND of AND delivery'' ) OR TITLE-ABS-KEY ( ''childbirth AND related AND fear'' ) OR TITLE-ABS-KEY ( ''prenatal AND fear AND of AND childbirth'' ) OR TITLE-ABS-KEY ( tokophobia ) OR TITLE-ABS-KEY ( tocophobia ) ) ) AND ( ( TITLE-ABS-KEY ( counselling ) OR TITLE-ABS-KEY ( intervention ) OR TITLE-ABS-KEY ( approache* ) OR TITLE-ABS-KEY ( *therapy ) OR TITLE-ABS-KEY ( psych* ) ) ) AND ( LIMIT-TO ( DOCTYPE , "re" ) ) AND ( LIMIT-TO ( LANGUAGE , "English" ) OR LIMIT-TO ( LANGUAGE , "Persian" ) ) AND ( LIMIT-TO ( SRCTYPE , "j" ) OR LIMIT-TO ( SRCTYPE , "p" ) )

**Cochrane**

#1 (''fear of childbirth''):ti,ab,kw OR (''fear of delivery''):ti,ab,kw OR (''childbirth related fear''):ti,ab,kw OR (''prenatal fear of childbirth''):ti,ab,kw OR (''postnatal fear of childbirth''):ti,ab,kw 749

#2 (tokophobia):ti,ab,kw OR (tocophobia):ti,ab,kw OR (''expectation of childbirth''):ti,ab,kw OR (''experience of childbirth''):ti,ab,kw 729

#3 #1 OR #2 1319

#4 (counselling):ti,ab,kw OR (intervention):ti,ab,kw OR (*therapy):ti,ab,kw OR (Psych*):ti,ab,kw OR (approache*):ti,ab,kw 1218266

#5 #3 AND #4 1120

**Web of Science**

1: ((((((((TS=(''fear of childbirth'')) OR TS=(tokophobia)) OR TS=(''fear of delivery'')) OR TS=(''childbirth related fear'')) OR TS=(''expectation of childbirth'')) OR TS=(''experience of childbirth'')) OR TS=(''prenatal fear of childbirth'')) OR TS=(''postnatal fear of childbirth'')) OR TS=(tocophobia) Results: 9643

2: ((((TS=(counselling)) OR TS=(intervention)) OR TS=(*therapy)) OR TS=(Psych*)) OR TS=(approache*) Results: 6214423

3: #1 AND #2 Results: 4529

4: ((TS=(review)) OR TS=(''systematic review'')) OR TS=(meta-analysis) Results: 2964401

5: #3 AND #4 Results: 864
